# Impact of left ventricular concentricity on long-term mortality in a hospital-based population in Japan

**DOI:** 10.1371/journal.pone.0203227

**Published:** 2018-08-30

**Authors:** Yuta Seko, Takao Kato, Yusuke Morita, Yuhei Yamaji, Yoshizumi Haruna, Toshiaki Izumi, Shoichi Miyamoto, Eisaku Nakane, Hideyuki Hayashi, Tetsuya Haruna, Moriaki Inoko

**Affiliations:** 1 Cardiovascular Center, The Tazuke Kofukai Medical Research Institute, Kitano Hospital, Osaka, Japan; 2 Department of Cardiovascular Medicine, Kyoto University Graduate School of Medicine, Kyoto, Japan; Indiana University, UNITED STATES

## Abstract

**Background:**

The prognostic impact of relative wall thickness (RWT), ventricular concentricity, is controversial.

**Methods:**

We retrospectively analyzed data obtained from 4444 consecutive patients who had undergone both transthoracic echocardiography and electrocardiography at our hospital in 2013. Those who presented with a history of previous episodes of myocardial infarctions and severe or moderate valvular disease were excluded from the analysis. We calculated RWT as follows: (2 x diastolic posterior wall thickness) / (the diastolic LV dimension). We defined high RWT as a ratio > 0.42. A total of 3654 patients were categorized into two groups: 492 with high RWT, and 3162 with normal RWT.

**Results:**

The mean ages of those in the normal and high RWT groups were 64.6 (±standard deviation 16.3) and 71.6 (± 12.7) years, respectively (p<0.001). Prevalence of male sex, history of diabetes, hypertension, and chronic kidney disease, and the left atrium volume index was higher for the high RWT group than for the normal RWT group. The median follow-up period was 1274 days (interquartile range, 410–1470). The Kaplan-Meier curves showed a constant increase in all-cause death, with cumulative 3-year incidences of 18.3% and 10.8% for the high RWT and normal RWT groups, respectively (log-rank p<0.001). After adjusting for confounders, the increased mortality risk for those with high RWT relative to normal RWT was significant (hazard ratio, 1.64; 95% confidence interval, 1.27–2.10). This trend was consistent for the composite of deaths and major adverse cardiac events.

**Conclusion:**

High RWT has a deleterious impact on long-term mortality.

## Introduction

Myocardial injury or overload usually causes left ventricular hypertrophy (LVH), which can be classified as eccentric or concentric, with or without changes in left ventricular (LV) function. LV dysfunction has been intensively investigated and established to link with poor clinical outcomes [1, 2]. However, the structural changes underlying the change of LV function were less investigated in terms of clinical outcomes [3]. LVH may be considered a physiological adaptation because the increasing LV wall thickness reduces LV wall stress and maintains cardiac output in hypertensive patients [4]. Patterns of LVH are usually classified with geometric remodeling that is determined by LV mass (LVM) and relative wall thickness (RWT). Increased LVM is associated with considerable cardiovascular morbidity and mortality in patients with hypertension and valvular heart disease or in the general population [5–8]. In contrast, the prognostic impact of the ratio of LV wall thickness to the chamber radius, is the definition of concentricity, which is referred to as RWT, is still controversial. In the present study, we tested the hypothesis that high RWT has a deleterious impact on long-term mortality in a hospital-based population in Japan.

## Methods

### Study population

**Fig 1:**
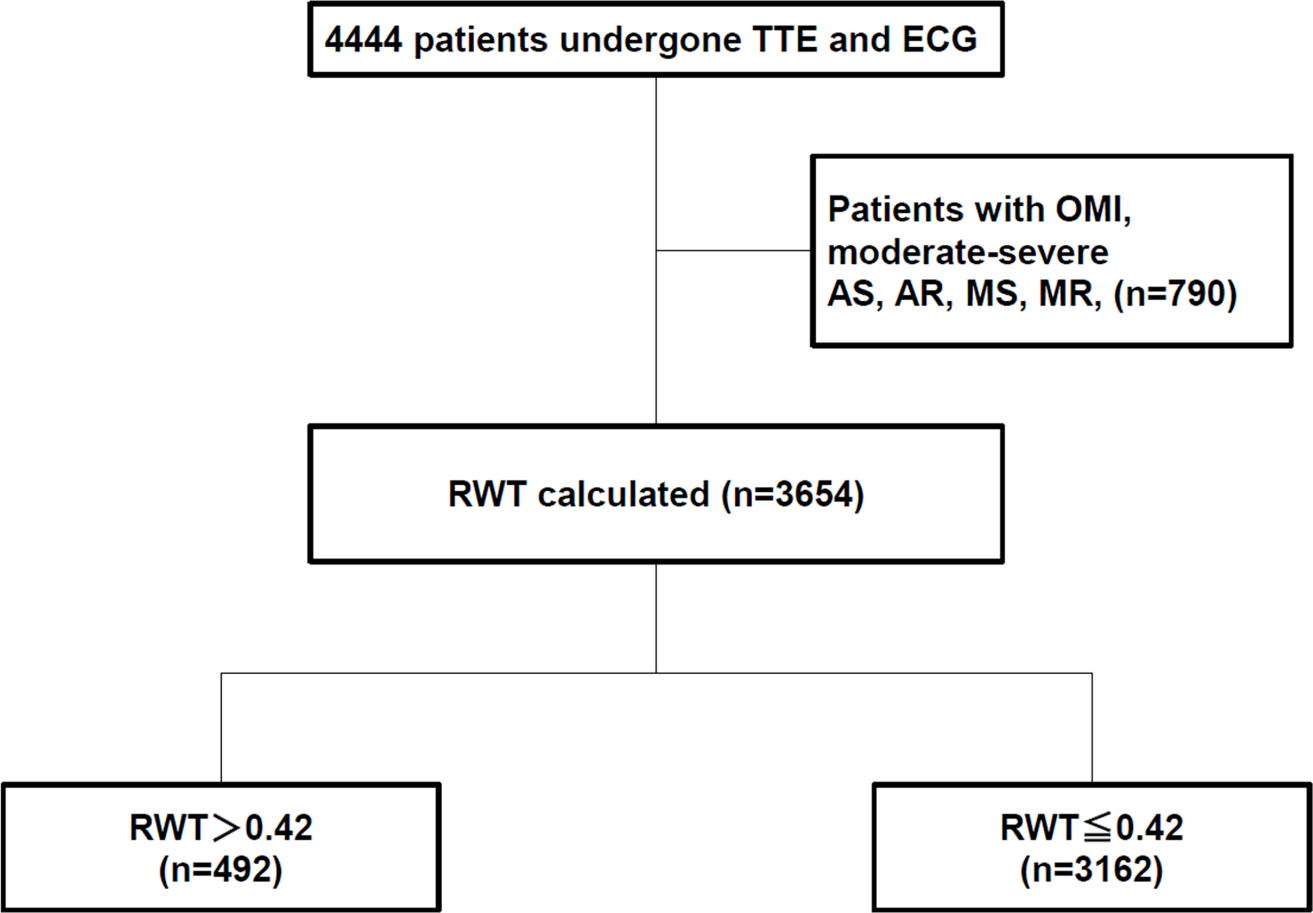
Flowchart of the study population. Abbreviations: TTE, transthoracic echocardiography; ECG, electrocardiography; LV, left ventricular; RWT, relative wall thickness; OMI, old myocardial infarction.

We retrospectively analyzed 4444 patients who had undergone simultaneous TTE and electrocardiography (ECG) at the Cardiovascular Center of Kitano Hospital during 2013 [9]. The ECG and TTE were ordered by each physician. A flowchart of the study population is shown in Fig 1. Moreover we A total of 790 patients who had findings of previous myocardial infarction (MI; N = 419) or severe or moderate valvular disease (aortic stenosis, N = 133; aortic regurgitation, N = 132; mitral stenosis, N = 9; and mitral regurgitation, N = 169) were excluded due to the effects on cardiac wall thickness. Based on the TTE and ECG data, and data from the catheter database, we identified patients who had a previous MI.

The research protocol was approved by the institutional review board of Kitano Hospital (approval number: P16-02-005). Informed consent was not obtained from each patient because this was a retrospective study. The study protocol conformed to the ethical guidelines of the 1975 Declaration of Helsinki, as reflected in a priori approval by the institution’s human research committee. Patients’ records and information were anonymized and de-identified before analysis. Data set was available in supporting information (S1 Dataset).

### Data collection

Using the TTE database, we extracted data regarding wall thickness, LV diastolic diameter (LVDd), left atrium diameter, left atrium volume index (LAVI), and LV ejection fraction (LVEF). We also extracted the body surface area data from the TTE report. From the ECG database, we extracted cardiac rhythm and recorded it as it was documented. Therefore, we could not determine whether atrial fibrillation (AF) was paroxysmal or persistent. RWT was calculated using the formula recommended by the American Society of Echocardiography (ASE) as follows: RWT: (2 × LVPWTd) / (LVDd), where LVPWTd was the diastolic LV posterior wall thickness. LV mass was calculated with the formula recommended by the American Society of Echocardiography (ASE) and was indexed to the body surface area as follows: LV mass = 0.8×1.04 [(LVDd + LVPWTd + IVSTd)3− (LVDd)3]+0.6, where LVDd was the LV diastolic diameter, IVSTd was the diastolic interventricular septal wall thickness and LVPWTd was the diastolic LV posterior wall thickness [10,11].

In line with the ASE recommendations, we defined high RWT as a ratio > 0.42. High LVMI was defined as LVMI >115 g/m2 for male patients and >95 g/m2 for female patients. The left atrium volume was calculated using the biplane area-length method and we defined the high left atrial volume as a value >42 mL/m^2^ [10]. Data from two-dimensional TTE were analyzed at baseline. LVEF was measured using the Teichholz method or the modified Simpson rule method.

We extracted the electronic patient medical data at our institution, including age, sex, and type of disease (i.e., ischemic heart disease, *International Statistical Classification of Diseases and Related Health Problems*, Tenth Edition [ICD-10] codes I20, I21, I22, I23, I24, and I25; hypertension, ICD-10 codes I10, I11, I12, I13, I14, and I15; dyslipidemia, ICD-10 code E78; diabetes mellitus, ICD-10 codes E10, E11, E12, E13, and E14; and chronic kidney disease, ICD-10 code N18). The follow-up data were also collected retrospectively in December 2016 from the serial clinical visits based on the electronic patient medical data.

### Outcome measures

The primary outcome measure was all-cause death. The secondary outcome measure was a composite of all-cause death and major adverse cardiac events (MACE) defined as acute heart failure, acute MI, unstable angina pectoris, cerebral infarction, cerebral hemorrhage, aorta and peripheral vascular disease including the treatment of aortic aneurysm. We also compared the primary and secondary outcomes among 4 LVH classifications.

### Statistical analysis

Categorical variables are presented as numbers and percentages. They were compared using the χ^2^ test or Fisher’s exact test. Continuous variables are expressed as mean (±standard deviation [SD]) or median (interquartile range [IQR]). Based on their distributions, the continuous variables were compared using the Student t-test or Wilcoxon rank-sum test. One-way ANOVA or Kruskal-Wallis test were used for the comparisons of 4 groups.

To analyze the factors associated with high RWT, we used a multivariable logistic regression model involving the following potentially independent clinically relevant variables: age, sex, body mass index, echocardiographic parameters, and comorbidities (Table 1).

**Table 1:** Baseline characteristics of the study subjects and transthoracic echocardiography results.

	Total (n = 3,654)	RWT≦0.42 (n = 3,162)	RWT>0.42 (n = 492)	p
*Age, yr, SD	65.5, 16.0	64.6, 16.3	71.6, 12.7	< .0001
*Male, %	52.5	51.7	57.7	0.012
*Diabetes, %	29.5	27.8	40.7	< .0001
*Hypertension, %	55.1	51.9	75.8	< .0001
*Dyslipidemia, %	28.6	26.6	36.6	< .0001
*Ischemic heart disease, %	25.4	24.3	32.3	0.0002
*Chronic kidney disease, %	13.2	11.5	24.6	< .0001
Body mass index, SD	23.1, 4.2	23.1, 4.1	23.4, 4.9	0.1995
*BMI>30, %	5.6	5.2	8.0	0.0126
LVDd, mm	46.3, 5.7	47.0, 5.5	42.1, 4.9	< .0001
LAD, mm	35.1, 6.6	34.9, 6.5	36.2, 7.1	< .0001
LAVI, ml/m2	23.1, 12.4	22.8, 12.3	24.9, 12.9	< .0001
*LAVI>0.42, %	5.5	5.0	8.7	0.0010
*EF, %	62.2, 6.9	62.2, 7.0	62.1, 5.8	0.7977
*AF, %	10.2	10.0	11.6	0.2882
*LVMI, g/m2	76.8, 23.9	75.0, 22.5	88.7, 28.8	< .0001

p Values were calculated from a χ2 test or Fisher’s exact test for categorical variables, and Student’s t-test or Wilcoxon rank sum test for continuous variables. Values are number (%), mean (SD) or median (IQR). BMI = Body mass index, LVDd = Left ventricular diastolic dimension, LAVI = Left atrial volume index, EF = Ejection fraction, AF = Atrial fibrillation, LVMI = Left ventricular mass index.

*Potential risk-adjusting variables selected for Cox proportional hazard models

Next, we compared the 3-year clinical outcomes between the high RWT and normal RWT groups. Cumulative incidences of clinical events were estimated using the Kaplan-Meier method, and the intergroup differences were assessed using the log-rank test. Multivariable Cox proportional hazards models were used to estimate the risk of high RWT relative to normal RWT and primary and secondary outcomes. The results were expressed as hazard ratios and 95% confidence intervals. We selected the clinically relevant risk-adjusted variables (Table 1) for the primary and secondary outcomes for use in the main analysis. Proportional hazard assumptions for the normal RWT and high RWT groups were assessed using plots of log (time) versus log [−log (survival)] stratified by variable and were verified as acceptable. We also evaluated the interactions between the subgroup factors and the effects of high RWT relative to normal RWT for clinical outcomes. Subgroup analyses of the primary outcome measures were also performed based on LVEF, hypertension, LV mass index, and AF at baseline.

Finally, we further categorized 3654 patients into four groups as follows (S1 Fig): high RWT and normal LVMI (n = 377), high RWT and high LVMI (n = 113), normal RWT and normal LVMI (n = 2880), normal RWT and high LVMI (n = 275). Nine patients without data on body surface area were excluded for analysis (S1 Fig). Cumulative incidences of clinical events were estimated using the Kaplan-Meier method and the intergroup differences were assessed using the log-rank test.

All statistical analyses were conducted by physicians (Y.S and T.K.) using JMP version 13 (SAS Institute Inc., Chicago, IL, USA). All reported p values were two-tailed, and p<0.05 was considered statistically significant.

## Results

### Baseline clinical and echocardiographic characteristics: Normal versus high RWT groups

A total of 492 patients had high RWT, and 3162 patients had normal RWT (Fig 1). The baseline characteristics of the entire study population are presented in Table 1. Patients in the high RWT group were older than those in the normal RWT group, were more often male, and were more likely to have hypertension, diabetes mellitus, dyslipidemia, ischemic heart disease, chronic kidney disease, smaller LV dimensions, and a higher LAVI (Table 1).

### Clinical outcomes: Normal versus high RWT groups

**Fig 2:**
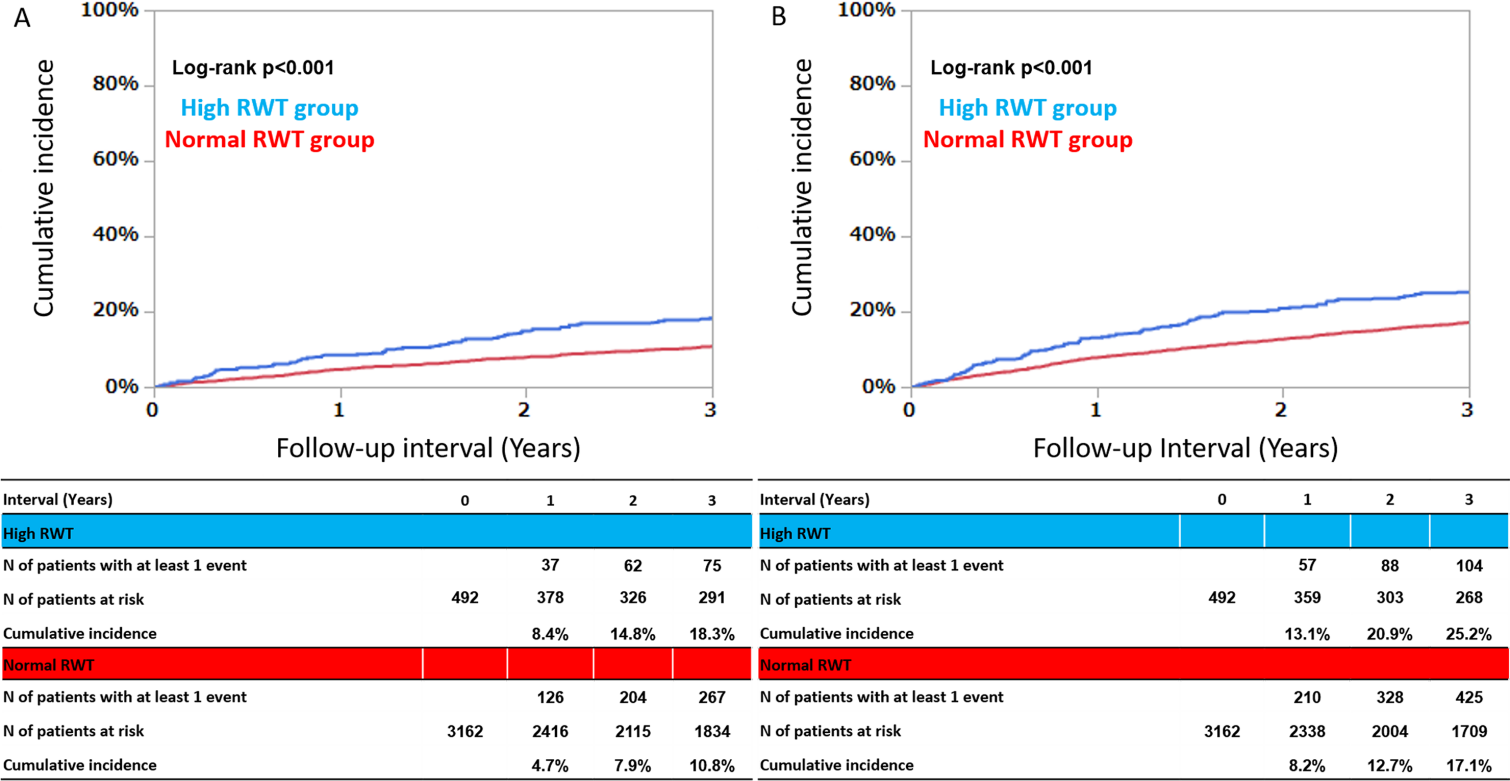
Primary and secondary outcomes. A) Cumulative incidence of the primary outcome measure (all cause death); normal versus high RWT groups. In the primary outcome, the cumulative 3-year incidences were significantly higher in the high RWT group than in the normal RWT group. After adjusting for confounders, the excess risk of high RWT relative to normal RWT for the primary outcome measure was remained significant. B) Cumulative incidence of the secondary outcome measure (all cause death or MACEs); normal versus high RWT groups. MACEs defined as acute heart failure, acute MI, unstable angina pectoris, cerebral infarction, cerebral hemorrhage, aortic dissection, and treatment of aortic aneurysm. In the secondary outcome, the cumulative 3-year incidences were significantly higher in the high RWT group than in the normal RWT group. After adjusting for confounders, the excess risk of high RWT relative to normal RWT for the secondary outcome measure was remained significant.

The median follow-up duration after the index echocardiography was 1274 days (IQR, 410–1470), with a follow-up rate of 80.9% at 1 year, 74.9% at 2 years, and 67.4% at 3 years. The cumulative 3-year incidences of the primary and secondary outcome measures were significantly higher for the high RWT group than for the normal RWT group (Fig 2A and 2B). After adjusting for confounders, the excess risk of high RWT relative to that of normal RWT for the primary and secondary outcome measures remained significant (Table 2).

**Table 2:** Clinical outcomes of patients in high and normal RWT groups.

	Normal RWT N of patients with at least 1 event (Cumulative 3-year incidence [%])	High RWT N of patients with at least 1 event (Cumulative 3-year incidence [%])	Unadjusted		Adjusted	
			HR (95% CI)	P value	HR (95% CI)	P value
	N = 492	N = 3162				
Primary endpoint: All-cause death	267 (10.8)	75 (18.3)	1.82 (1.44–2.29)	<0.001	1.64 (1.27–2.10)	0.0002
Secondary endpoint: All-cause death or MACEs	425 (17.1)	104 (25.2)	1.84 (1.43–2.09)	<0.001	1.34 (1.08–1.65)	0.0073
Heart Failure	82 (3.6)	16 (4.2)	1.54 (0.95–2.38)	0.076	1.03 (0.60–1.67)	0.91
Unstable Angina Pectoris	18 (0.7)	2 (0.5)	1.06 (0.25–3.13)	0.93	0.49 (0.11–1.55)	0.24
Myocardial Infarction	8 (0.3)	3 (0.8)	4.80 (1.58–13.8)	0.0072	3.04 (0.87–9.64)	0.079
Aorta and peripheral vascular disease	29 (1.2)	3 (0.4)	1.12 (0.42–2.48)	0.80	0.73 (0.27–1.70)	0.49
Cerebral Infarction	40 (1.7)	13 (3.5)	2.11 (1.16–3.63)	0.015	1.52 (0.79–2.79)	0.20
Cerebral Hemorrhage	16 (0.7)	2 (0.6)	0.96 (0.23–2.79)	0.94	0.83 (0.19–2.60)	0.77

### Subgroup analysis: Normal versus high RWT groups

**Fig 3:**
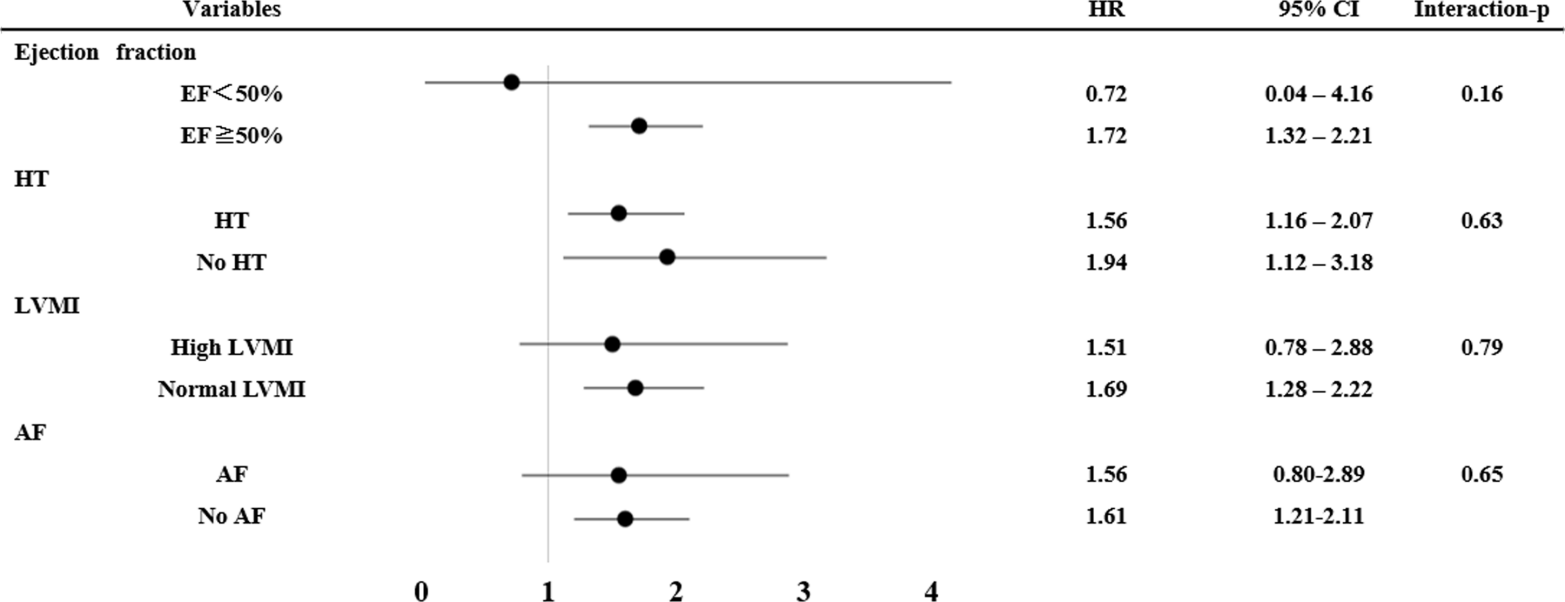
Subgroup analyses. HR = Hazard rate, CI = Confidence interval, EF = Ejection fraction, HT = Hypertension, LVMI = Left ventricular mass index, AF = Atrial fibrillation.

According to the subgroup analyses stratified by LVEF, hypertension, LV mass index, and AF, there were no significant interactions between the subgroup factors and the effects of high RWT for the primary outcome measures (Fig 3). Furthermore, when classified into 4 groups of LV hypertrophy using additional LVMI data (S1 Table), the cumulative 3-year incidences of the primary outcome measures were consistent with the main analysis (S2 Fig).

## Discussion

The main findings of this study are as follows: 1) Patients in the high RWT group were older, more often male, and were more likely to have comorbidities, smaller LV dimensions, and a higher LAVI; 2) a high RWT had a deleterious impact on the outcomes of patients in our study population; 3) there were no interactions between the effect of a high RWT and ejection fraction, hypertension, a high LVMI, or atrial fibrillation.

There have been few studies, especially in Japanese population, that focus on the increase in chamber radius, an adaptation of the cardiac structure, in which RWT is increased [12,13]. In our study, patients with high RWT were characterized as being older and with more comorbidities and diastolic dysfunction than those with normal RWT. After adjusting for confounders, a high RWT was still an independent factor associated with worse outcomes, for which there might be three possible reasons. First, the decreased elastane of the LV has been shown to be associated with an increase in compliance of the arteries [1,14]. Increased afterloads may be linked to increased MACEs such as acute heart failure and aortic disease. Second, increased RWT has been reported to be associated with high levels of epinephrine, aldosterone, and hepatocyte growth factor. The mechanistic link is not clear; however, these factors can contribute to increased cardiovascular events [15]. Third, the coronary flow reserve may be limited in patients with a high RWT. There was a report that in hypertensive patients, coronary flow reserve may be impaired due to humoral disturbances such as hyperglycemia, hyperinsulinemia, and hyperaldosteronism [16, 17]. In addition, the sub-endomyocardial tissue may be vulnerable to ischemia with an increase in chamber radius because of the blood supplied inward from the epicardial region [18].

This is the first report showing the impact of RWT on outcomes in a hospital-based population in Japan with 3-year follow-up; however, there were conflicting results regarding the prognostic impact of a high RWT. Ghali et al. reported that both high LVMI and RWT had the highest mortality, but concentric remodeling (high RWT and normal LVMI) was not associated with an increased risk of death in patients with suspected coronary artery disease [8]. As mentioned above, the patients with concentric remodeling probably associated with an increased risk of mortality because they have high RWT. In our study, the patient with concentric remodeling (high RWT and normal LVMI) still had a worse primary outcome. Milani et al. also reported that high RWT has been shown to be associated with mortality and poor cardiovascular outcomes in a hospital-based population [12]. Verma et al. also reported the increased risk associated with RWT independently of LVMI in patients with MI [13]. The results of our study are in line with the studies of Milani and Verma. The noted difference between the studies of Milani and Verma and our study was the patient population analyzed. Due to the difference in ethnicity, the patients in our study had a smaller body mass index. We included patients with coronary artery disease but excluded patients with MI because the calculation of RWT was influenced by the infarcted wall thickness. In addition, the report of Milani and Verma excluded patients with low EF (EF<45% and <50%, respectively) [8,12]. In the present study, we included these patients but adjusted by LVEF when analyzed. The subgroup analyses were performed according to the LV function, the presence of hypertension, a high LVMI, and the presence of AF. Regardless of LV dysfunction, a high LVMI, or the presence of AF, a high RWT was related to the primary outcome in the present study. Our results implied that, even in the different races and backgrounds, the structural changes of left ventricle had a deleterious impact. The clinical implication of the present study is as follows. The structural change has a deleterious impact; therefore, the underlying causes of the structural LV change should be carefully observed or be treated.

### Limitations

This study had several limitations. First, the ordering criteria for ECG and TTE were not set. Second, patient data were extracted from electronic medical data, respectively. This resulted in low follow-up rate, especially at 3 years. Third, data regarding NYHA functional class or brain natriuretic peptides levels were not obtained in the present study. Fourth, this was a single-center study in Japan; thus, possible selection bias could not be excluded despite the large sample size. Finally, there remain unmeasured confounders affecting the long-term prognosis, although we conducted extensive statistical adjustment for the measured confounders.

## Conclusion

Patients with high RWT are at higher long-term risk for clinical events.

## Supporting information

S1 Dataset Dataset containg the values used in this study. (XLSX)

S1 Fig Flowchart of the study population according to 4 classification. Abbreviations: RWT, relative wall thickness; LVMI, left ventricular mass index.
(DOCX)

S2 Fig Cumulative incidence of the primary outcome measure: High RWT and Normal LVMI, High RWT and High LVMI, Normal RWT and High LVMI, and Normal RWT and Normal LVMI groups. (DOCX)

S1 Table Baseline characteristics of the study subjects and transthoracic echocardiography results according to 4 classification. (DOCX)
