# Supplementary figures and images for: Impact of left ventricular concentricity on long-term mortality in a hospital-based population in Japan
Source: PLoS One. 2018 Aug 30;13(8):e0203227. doi: 10.1371/journal.pone.0203227 (PMC6117041; doi:10.1371/journal.pone.0203227)

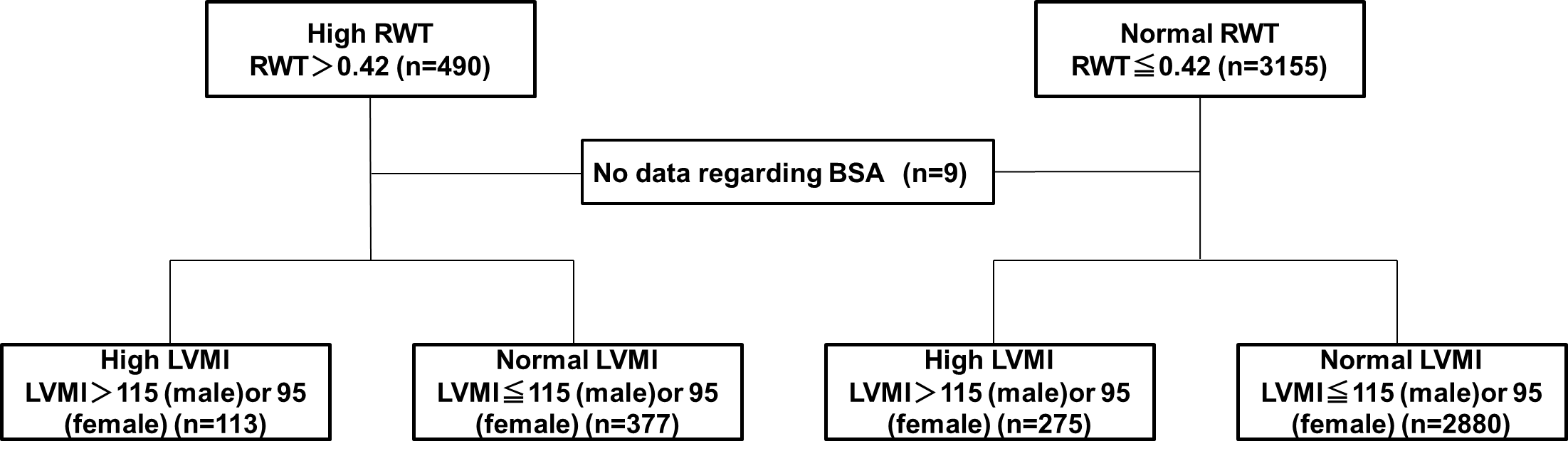

Supplement: S1 Fig — Abbreviations: RWT, relative wall thickness; LVMI, left ventricular mass index. (DOCX) [file pone.0203227.s002.docx]

**
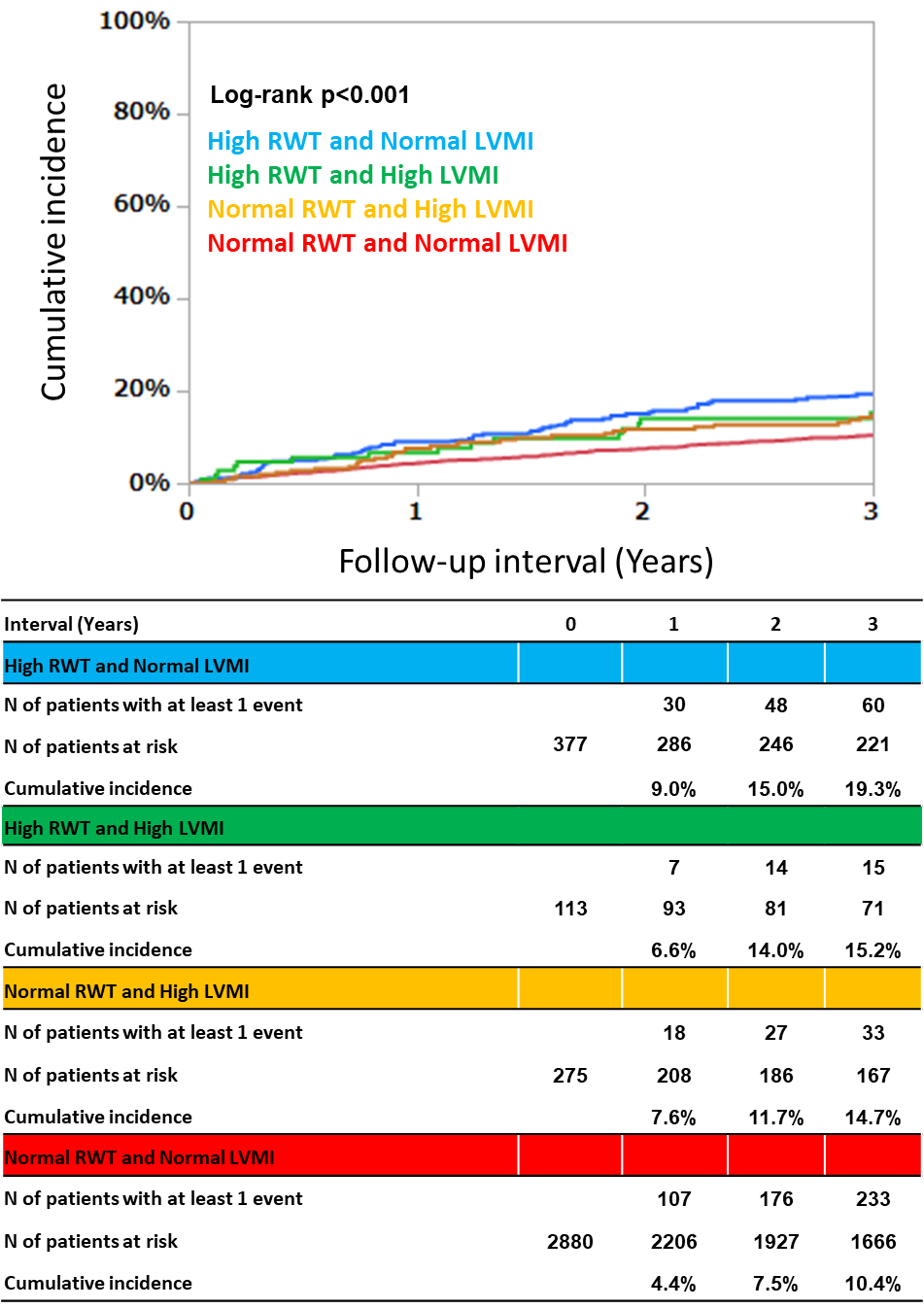
**

Supplement: S2 Fig — (DOCX) [file pone.0203227.s003.docx]
