# Supplementary material for: Impact of left ventricular concentricity on long-term mortality in a hospital-based population in Japan
Source: PLoS One. 2018 Aug 30;13(8):e0203227. doi: 10.1371/journal.pone.0203227 (PMC6117041; doi:10.1371/journal.pone.0203227)
Supplement: S1 Table — (DOCX) [file pone.0203227.s004.docx]

|  | Normal RWT  (n=3,162) | | High RWT  (n=492) | |  |
| --- | --- | --- | --- | --- | --- |
|  | Normal LVMI (n=2,880) | High LVMI (n=275) | Normal LVMI (n=377) | High LVMI (n=113) | p |
| Age, yr, SD | 64.0, 16.4 | 69.8, 13.6 | 71.9, 12.4 | 70.6, 13.7 | ＜.0001 |
| Male, % | 52.6 | 43.3 | 60.0 | 51.3 | 0.0005 |
| Diabetes, % | 27.0 | 36.4 | 39.0 | 46.9 | ＜.0001 |
| Hypertension, % | 49.7 | 76.0 | 72.4 | 88.5 | ＜.0001 |
| Dyslipidemia, % | 26.3 | 38.2 | 35.8 | 40.7 | ＜.0001 |
| Ischemic heart disease, % | 23.3 | 35.3 | 31.6 | 35.4 | ＜.0001 |
| Chronic kidney disease, % | 10.2 | 25.1 | 19.9 | 40.7 | ＜.0001 |
| Body mass index, SD | 23.0, 4.0 | 23.8, 5.0 | 23.3, 5.0 | 23.5, 4.4 | 0.0633 |
| BMI＞30, % | 4.8 | 8.7 | 8.0 | 8.0 | 0.0038 |
| LVDd, mm | 46.3, 4.7 | 54.6, 7.1 | 40.8, 4.1 | 46.4, 4.8 | ＜.0001 |
| LAD, mm | 34.4, 6.3 | 40.3, 7.0 | 35.1, 6.8 | 39.6, 6.9 | ＜.0001 |
| LAVI, ml/m2 | 21.8, 11.6 | 32.8, 14.2 | 22.9, 11.8 | 31.5, 13.9 | ＜.0001 |
| LAVI＞0.42, % | 3.8 | 17.2 | 5.5 | 18.6 | ＜.0001 |
| EF, % | 62.9, 5.7 | 54.9, 13.2 | 62.5, 5.0 | 60.5, 7.8 | ＜.0001 |
| AF, % | 9.6 | 14.2 | 10.9 | 13.3 | 0.0623 |

p Values were calculated from a χ2 test or Fisher’s exact test for categorical variables, and Student’s t-test or Wilcoxon rank sum test for continuous variables. Values are number (%), mean (SD) or median (IQR). BMI=Body mass index, LVDd=Left ventricular diastolic dimension, LAVI=Left atrial volume index, EF=Ejection fraction, AF=Atrial fibrillation, LVMI=Left ventricular mass index.
